# Schistosomal appendicitis: Case series and systematic literature review

**DOI:** 10.1371/journal.pntd.0009478

**Published:** 2021-06-24

**Authors:** Mateus Zacarias, Damiano Pizzol, Helder de Miranda, Anna Claudia Colangelo, Nicola Veronese, Lee Smith

**Affiliations:** 1 Department of Surgery, Central Hospital of Beira, Beira, Mozambique; 2 Italian Agency for Development Cooperation, Khartoum, Sudan; 3 Department of Surgery and Organ Transplantation, University of Padua, Padua, Italy; 4 Geriatric Unit, Department of Internal Medicine and Geriatrics, University of Palermo, Palermo, Italy; 5 The Cambridge Centre for Sport & Exercise Sciences, Anglia Ruskin University, Cambridge, United Kingdom; George Washington University School of Medicine and Health Sciences, UNITED STATES

## Abstract

**Background:**

Globally, schistosomiasis affects at least 240 million people each year with a high proportion of cases in sub-Saharan Africa. The infection presents a wide range of symptoms mainly at the gastrointestinal and urogenital level. Cases of schistosomiasis-related appendicitis are seldom reported. The aim of the present study is to identify the prevalence of schistosomiasis-related appendicitis in Beira, Mozambique and compare to global prevalence.

**Methods:**

We retrospectively reviewed all cases of appendicitis recorded from January 2017 to March 2020 at a single pathology department located in Beira in order to assess the prevalence of schistosomiasis. Moreover, we performed a systematic review on the prevalence of schistosomiasis-related appendicitis in all countries.

**Findings:**

A total of 145 appendicitis cases in Beira showed a 13.1% prevalence of schistosomal-related appendicitis. The mean age of patients was 29.1 years, and 14 (73.7%) were male. The systematic review identified 20 studies with 34,790 inpatients with schistosomiasis-related appendicitis with a global prevalence of 1.31% (95% confidence interval (CI): 0.72 to 2.06); a high heterogeneity (I^2^ = 96.0%) was observed. Studies carried out in Africa reported a significantly higher prevalence of schistosomiasis-related appendicitis (2.75%; 95% CI: 1.28 to 4.68) than those in Middle East (0.49%; 95% CI: 0.18 to 0.95) (*p* for interaction < 0.0001).

**Conclusions:**

Schistosomiasis infection should be considered as possible cause of appendicitis not only in endemic areas but also in developed countries. Considering that prevention is the best way to control the infection, more efforts should be put in place in order to increase the prevention coverage and avoid the cascading implications for health. This is even more so important in this Coronavirus Disease 2019 (COVID-19) era where the majority of attention and funds are used to fight the pandemic.

## Author summary

Schistosomiasis is a parasitic waterborne disease affecting more than 240 million people worldwide every year, especially in sub-Saharan Africa. The infection leads to different grades of illness at the gastrointestinal and urogenital level. Acute appendicitis is seldom reported as schistosomal consequence, but the prevalence is underestimated. In our study, including 145 patients, we reported a 13% prevalence of parasitic infection; this is 10 times higher than what was identified in the systematic review including almost 35,000 people and showing a total prevalence of 1.31%. It will be crucial to strengthen the prevention coverage in order to avoid the cascading implications for health, especially in this Coronavirus Disease 2019 (COVID-19) era where the majority of attention and funds are used to fight the pandemic.

## Introduction

One of the main public health issues in tropical and subtropical regions worldwide is represented by parasitic infestations, and it is estimated that more than 880 million children are in need of treatment for these parasites [1]. Schistosomiasis is a parasitic waterborne disease caused by blood flukes of the genus *Schistosoma* [2]. Six species infect humans, namely *Schistosoma guineensis*, *Schistosoma haematobium*, *Schistosoma intercalatum*, *Schistosoma japonicum*, *Schistosoma mansoni*, and *Schistosoma mekongi*. Although all species are able to cause disease, the 3 predominant are *S*. *haematobium*, *S*. *mansoni*, and *S*. *japonicum*. Schistosomiasis affects at least 240 million people worldwide, with about 20 million people with severe and debilitating illness every year [3]. The prevalence of schistosomiasis is highest in sub-Saharan Africa and, according to the World Health Organization (WHO), the estimated total number of people who needed treatment for schistosomiasis in 2014 was almost 260 million, of which more than 123 million (47.6%) were school-aged children (5 to 14 years old) [4,5]. Moreover, in 2018, WHO estimated that 229 million people needed treatment for schistosomiasis [6]. Interestingly, it was estimated that in 2014, 91.4% of these people lived in Africa [5]. In Mozambique, according to an epidemiological survey among school-aged children between 2005 and 2007, schistosomiasis is a major public health problem [7]. The infection occurs by skin penetration, and the most affected systems are gastrointestinal and urogenital [2]. Although the intestinal infestation can present with a wide range of symptoms considering that any part of the gastrointestinal tract can be affected, rarely, it causes appendicitis [8].

In general, parasitic infections of appendix are not associated with an acute inflammation, but the presence of schistosomiasis, especially the species *S*. *haematobium*, *S*. *mansoni*, and *S*. *japonicum*, in the appendix may lead to lumen obstruction causing appendiceal colic [9]. Schistosomiasis represents an uncommon granulomatous inflammatory appendicitis, and its differential diagnosis includes tuberculosis, *Yersinia*, and Crohn disease [10]. The pathophysiologic mechanism involves both the host’s immune system reaction to the schistosome eggs and the granulomatous inflammation induced by the antigens secreted by parasites [10]. Although the granulomas are able to destroy the eggs, the inflammatory process results in tissue fibrosis, and, depending on intensity and duration, the severity of disease is determined [10]. Based on these processes, 2 pathways leading to acute appendicitis have been described. The direct mechanism includes an immunological granulomatous reaction due to the eggs deposition that leads to the tissue destruction and, thus, to acute appendicitis [11]. In the indirect mechanism, chronic inflammation and calcified eggs result in massive fibrosis resulting in obstruction followed by bacterial infection [11]. In the case series, calcified eggs were identified in only one case, suggesting that the direct mechanism is more often involved in these appendicitis.

The clinical presentation of patients with appendicular schistosomiasis is a typical picture of acute appendicitis, and it represents a surgical emergency. Considering the a-specific signs and symptoms that may appear in patients affected by schistosomiasis, especially in endemic areas, health workers should pay particular attention to common diseases such as tuberculosis and malaria [12,13]. This is particularly important in this era of Coronavirus Disease 2019 (COVID-19) pandemic considering the so-called “syndemic” effect [13]. The differential diagnosis only can be made by histopathologic examination that shows schistosomal eggs with lateral spine and granulomatous inflammation as pathognomonic findings [14]. Blood tests are occasionally useful in supporting the diagnosis or assessing the severity of schistosomal infection, while serologies and polymerase chain reaction (PCR) assay can confirm the diagnosis. Urinary microbiology is instead crucial for diagnosing vesicular infection and faecal microbiology for primary bowel infection [15].

Considering the role that schistosomiasis can play in appendicitis and the literature gap in this field, the aim of the present study is to identify the prevalence of schistosomiasis-related appendicitis in Beira, Mozambique and compare to the global prevalence. In this article, we present a case series from 2017 to 2020 based on pathological findings at the Central Hospital of Beira (CHB) and perform a systematic review on the prevalence of schistosomiasis in appendicitis.

## Methods

### Case series

#### Ethics statement

The Clinical Board of Beira Central Hospital approved the study and granted the use of anonymised data for scientific purposes in June 2020. The Clinical Board waived the need for written informed consent given the retrospective nature of the study and the use of anonymised data from hospital records.

#### Setting

The city of Beira has approximately 500,000 inhabitants, of which 17% are less than 5 years. The CHB is a 1,020-bed government tertiary referring and teaching hospital for the central region of the country (population approximately 7 million) in Mozambique and the second hospital in the country. The CHB Department of Pathology consists of 4 specialists and is a landmark for the whole city of Beira and Province of Sofala.

#### Data collection and analysis

Data registers of CHB’s Department of Pathology were retrospectively reviewed to identify all cases of appendicitis recorded from January 2017 to March 2020. The extracted data provided a database with general information, organised in the following variables: presence of schistosomiasis, chronic condition, gender, and age. We conducted a descriptive analysis on all collected data.

#### Systematic review

This systematic review adhered to the PRISMA [16] and MOOSE [17] statements and followed a preplanned protocol (S1 Protocol).

#### Search strategy

Two investigators (NV and DP) independently conducted a literature search using MEDLINE/PubMed, Scopus, CINAHL, Embase, PsycINFO, and Cochrane Library databases from inception on 5 July 2020. The following search strategy was used: “Schistosomiasis” OR “Bilharzia” OR “Schistosoma” OR “*Schistosoma mansoni*” OR “Schistosoma haematobium” OR “Schistosoma japonicum” OR “Schistosoma mekongi” OR “Schistosoma guineensis” OR “Schistosoma intercalatum” OR “Schistosome” OR “Blood flukes” OR “Trematode” OR “Trematoda” OR “Trematode infections” OR “Trematode worms,” AND “Appendicitis” OR “Appendectomy” OR “Appendicectomy.” The references of retrieved articles together with the proceedings of relevant conferences were hand searched in order to identify other potentially eligible studies for inclusion in the analysis missed by the initial search or any unpublished data.

The literature search, assessment of inclusion and exclusion criteria, quality of studies, and extraction of data were independently undertaken and verified by 2 investigators (ZM and DP). The results were then compared, and, in case of discrepancies, a consensus was reached with the involvement of a third senior investigator (LS). There was no language restriction.

### Type of studies, inclusion, and exclusion criteria

Following the participants, intervention, controls, outcomes, study (PICOS) design criteria, we included studies assessing the following:

P: People with appendicitis;
I: None;
C: None;
O: Number/prevalence of schistosomiasis;
S: Observational (case–control and cross-sectional).

All retrospective or prospective studies reporting the prevalence of schistosomiasis in appendicitis were included. Studies were excluded if they had no data on prevalence of schistosomiasis or if it was related to other gastrointestinal disease. No language restriction was placed.

### Data extraction and statistical analyses

For each eligible study, 2 independent investigators (NV and DP) extracted the following: name of the first author and year of publication, setting, sample size, mean age of the population, % of females, % acute appendectomy, % of patients with nausea, vomiting, pain in right iliac fossa, tender in right iliac fossa, abdominal guarding, and fever.

#### Outcomes

The primary outcome was the prevalence of people having a diagnosis of schistosomiasis in those having a diagnosis of appendicitis.

#### Assessment of study quality

Two independent authors (ZM and ACC) assessed the quality of studies using the Newcastle–Ottawa scale (NOS) [18]. The NOS assigns a maximum of 9 points based on 3 quality parameters: selection, comparability, and outcome. As per the NOS grading in past reviews, we graded studies as having a high (<5 stars), moderate (5 to 7 stars), or low risk of bias (≥8 stars) [19].

#### Data synthesis and statistical analysis

All analyses were performed using Stata, version 15.0. For all analyses, a *p*-value less than 0.05 was considered statistically significant.

The primary analysis reported the prevalence (%) of schistosomiasis in people having appendicitis, with its 95% confidence intervals (CIs). Heterogeneity across studies was assessed by the I^2^ metric. Where significant heterogeneity was observed (I^2^ ≥ 50% and/or *p* < 0.05) [20], meta-regression analyses were run, taking as moderators the factors stated in the data extraction in the sample as whole.

Publication bias was assessed by visual inspection of funnel plots and using the Egger bias test [21]. In case of publication bias, when ≥3 studies were available, we used the Duval and Tweedie nonparametric trim-and-fill method to account for potential publication bias [22]. Based on the assumption that the effect sizes of all the studies are normally distributed around the centre of a funnel plot, in the event of asymmetries, this procedure adjusts for the potential effect of unpublished (trimmed) studies [21].

## Results

### Case series

A total of 145 appendicitis cases were registered at the Department of Pathology at the CHB between January 2017 and March 2020 (Table 1). Nineteen (13.1%) were diagnosed as having schistosomal-related appendicitis, and among them, 14 (73.7%) were male. The mean age was 29.1 years, and 13 (68.4%) had a chronic condition. In all samples, schistosomal ova was found except in one case where calcified ova was identified.

**Table 1:** Case series of schistosomal appendix at Beira Hospital from 2017 to 2020.

	2017	2018	2019*	2020**	Total
***N* appendicitis**	50	66	22	7	145
**Schistosomiasis *N* (%)**	7 (14)	9 (13.6)	1 (4.5)	2 (28.6)	19 (13.1)
**Sex male/female (%)**	5/2 (71.4)	8/1 (88.9)	0/1 (0)	1/1 (50)	14/5 (73.7)
**Mean age (range)**	32.6 (22–57)	25.4 (19–50)	26	35 (28–42)	29.1 (19–57)
**Chronic *N* (%)**	4 (57.1)	9 (100)	0 (0)	0 (0)	13 (68.4)

*The low number of cases was due to the Idai Cyclone and the interruption of many activities including suspension of surgeries.

**Data are referred until 31 March 2020, before COVID-19 emergency.

COVID-19, Coronavirus Disease 2019.

### Systematic review

#### Literature search

**Fig 1:**
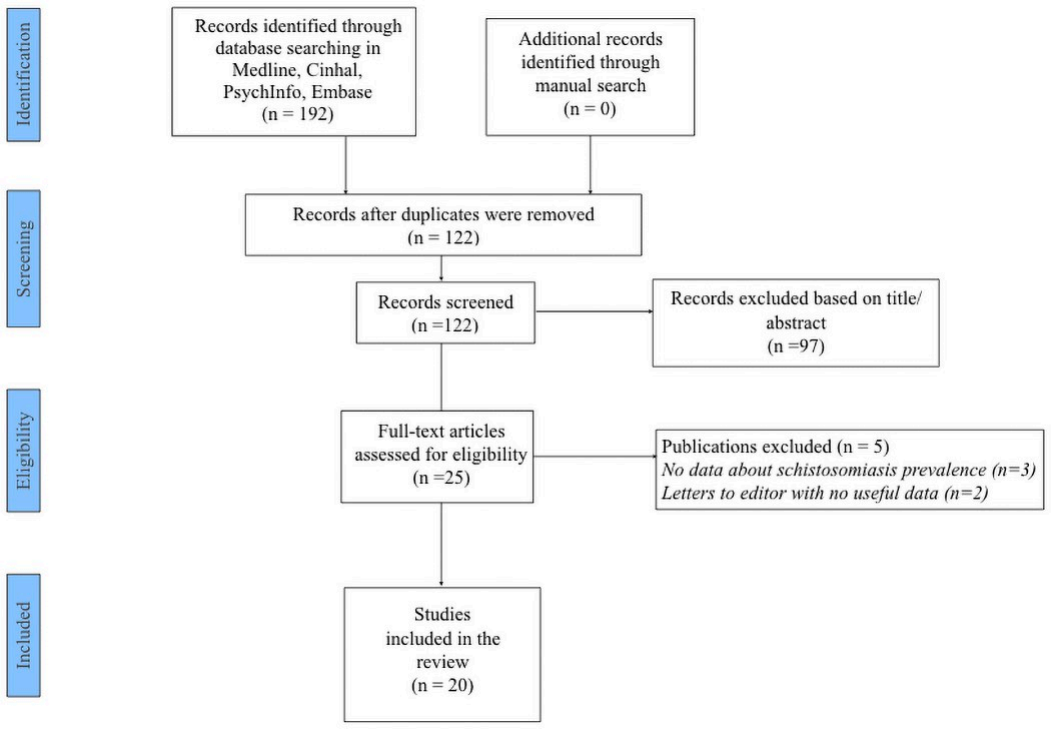
PRISMA flowchart.

As shown in Fig 1, we initially found 122 possible eligible articles. After removing 97 papers through the title/abstract screening, 25 were retrieved as full text. Of the 25 full text, 20 satisfied the inclusion/exclusion criteria and were included in the present systematic review and meta-analysis.

#### Excluded studies

Among the relevant studies, 5 failed to meet the inclusion criteria and were excluded from this review owing to lack of data on schistosomiasis prevalence. Moreover, 2 identified articles were letters to the editor that did not report appropriate data.

### Descriptive data

The 20 cross-sectional studies included a total of 34,790 inpatients with appendicitis. The mean age of participants was 23 years (even if the information was reported in only 4/20 studies included) and were mainly male (mean percentage of females = 37.1%) (Table 2).

**Table 2:** Descriptive findings included studies.

Author(s), year	Country	Continent	Sample size	Number of schistosomiasis	Mean age	% of females	NOS
Adebamowo and colleagues (1991) [23]	Nigeria	Africa	627	15	NR	NR	7
Adisa and colleagues (2009) [24]	Nigeria	Africa	956	22	NR	NR	6
Ahmed and colleagues (2014) [25]	Nigeria	Africa	1,464	30	NR	NR	5
Ahmed and colleagues (2017) [26]	Egypt	Africa	30	1	NR	0	6
Amer and colleagues (2017) [27]	Egypt	Africa	65	1	NR	29.2	9
Badmos and colleagues (2006) [28]	Nigeria	Africa	843	35	NR	NR	7
Botes and colleagues (2015) [29]	South Africa	Africa	304	31	NR	48	6
Duvie and colleagues (1987) [30]	Nigeria	Africa	518	32	NR	NR	5
Elfaedy and colleagues (2019) [31]	Libya	Africa	4,012	8	23	54	7
Hedya and colleagues (2012) [32]	Egypt	Africa	251	3	22.2	NR	7
Hodasi (1988) [33]	Ghana	Africa	2,584	76	NR	NR	5
Nandipati and colleagues (2008) [10]	USA	America	1,690	3	NR	NR	5
Abo-Alhassan and colleagues (2016) [34]	Kuwait	Middle East	3,012	8	NR	NR	6
Abu-Eshy and colleagues (1994) [35]	Saudi Arabia	Middle East	4,708	64	NR	NR	8
Al-Kraida and colleagues (1988) [36]	Saudi Arabia	Middle East	1,920	15	NR	NR	5
Dincel and colleagues (2017) [37]	Turkey	Middle East	1,970	2	NR	NR	6
Karatepe and colleagues (2009) [38]	Turkey	Middle East	5,100	6	24.9	34.7	6
Satti and colleagues (1987) [39]	Saudi Arabia	Middle East	1,600	26	NR	NR	5
Zaghlool and colleagues (2015) [9]	Saudi Arabia	Middle East	1,536	2	22.2	38.9	6
Zakaria and colleagues (2012) [40]	Saudi Arabia	Middle East	1,600	8	NR	54.8	7

The studies were mainly carried out in Africa (11 studies), followed by the Middle East (*n* = 8).

NOS, Newcastle–Ottawa scale; NR, Not reported.

### Prevalence of schistosomiasis in appendicitis

**Fig 2:**
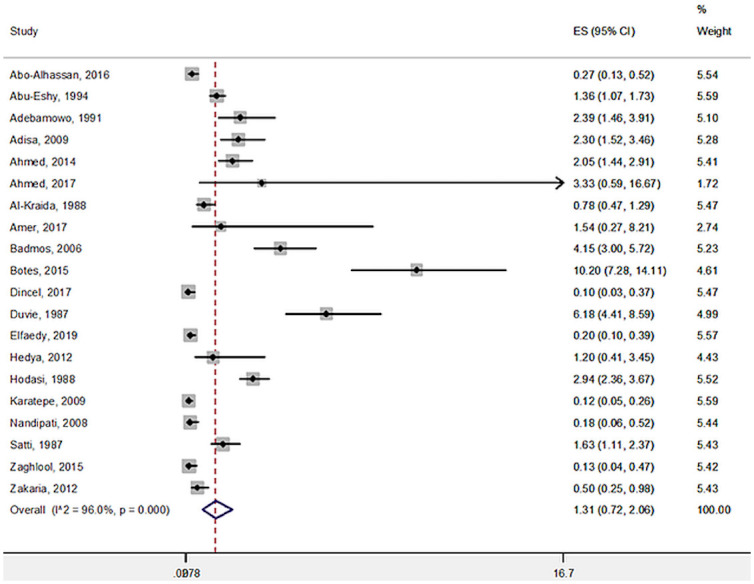
Prevalence of schistosomiasis in appendicitis. CI, confidence interval; ES, Effect Size.

Overall, in 20 studies with 34,790 inpatients with appendicitis, 388 had a diagnosis of schistosomiasis, leading a total prevalence of 1.31% (95% CI: 0.72 to 2.06) (Fig 2), with a high heterogeneity (I^2^ = 96.0%). After stratifying by continent, studies in Africa reported a significant higher prevalence of schistosomiasis (2.75%; 95% CI: 1.28 to 4.68) than the studies carried out in Middle East (0.49%; 95% CI: 0.18 to 0.95) (*p* for interaction < 0.0001).

Due to high heterogeneity of the primary outcome, several meta-regression analyses were carried out. However, as shown in Table 3, no moderator was able to explain the high heterogeneity found.

**Table 3:** Meta-regression analysis.

Moderator	Number of comparisons	Beta	Standard error	*p*-value
**% of females**	7	0.0002	0.009	0.89
**Mean age**	4	−0.0005	0.009	0.96
**% of acute appendectomy**	8	−0.0005	0.0006	0.40
**% of pain in right iliac fossa**	8	−0.001	0.0009	0.30
**% of tender in right iliac fossa**	3	−0.00009	0.002	0.96
**% of fever**	5	0.0004	0.0005	0.52

### Publication bias

No included outcome suffered on publication bias.

### Risk of bias

The risk of bias, evaluated through the NOS, is fully reported in Table 2 (as total score) and S1 Table in detail. The median quality of the studies was 6.2 (range: 5 to 9), indicating an overall more than satisfactory quality of the included studies. Specifically, one was a very good quality study (9 points), 6 were good (7 to 8), and 13 were satisfactory (5 to 6).

## Discussion

Acute appendicitis caused by schistosomiasis is considered a very rare event also in endemic areas. In our study, based on pathological findings and including 145 patients, we reported a 13% prevalence of parasitic infection. This rate is 10 times higher than the prevalence we found in the present systematic review including almost 35,000 people, showing a total prevalence of 1.31%. We believe that the present primary data are better reflecting the situation of endemic areas where it is likely that the incidence of schistosomal infection has previously been underestimated. In fact, the diagnosis can only be confirmed by histology of the removed appendix, and this is not often possible in developing countries especially in the most rural regions. In support of this hypothesis, data from this systematic review showed a significant higher prevalence of schistosomiasis (2.75%; 95% CI: 1.28 to 4.68) in studies performed in Africa compared to those in the Middle East that represent the 2 main settings of existing research in this area. The majority of affected patients in the present primary data were male (73.7%). Indeed, this is in line with the literature reporting a mean percentage of females of 37.1%. It is not clear if sex has a key role as a facilitating factor, and no hypothesis have been raised to date. As reported in the general literature, also in the present case series, the most affected decade was the 1920s, and, specifically, we reported a mean age of 29 years while the patients from the review aged a mean of 23 years.

Regardless the pathophysiology, the acute appendicitis represents a surgical emergency, and, considering the continuous changes in global migration, it should be taken into account also in developed countries as recently demonstrated [10]. In order to avoid the presentation of such potentially lethal manifestations, large-scale prevention, by anthelmintic praziquantel administration, is the most effective and efficacious strategy. This represents also the goal of WHO that aims to reduce schistosomiasis morbidity by 2020 and to eliminate schistosomiasis as a public health burden by 2025 [41].

The combination of a novel case series and meta-analyses is a clear strength of this study. However, findings from this study should be interpreted in light of its limitations. First, the small sample size and the fact that there was partial data of the case series mainly due to the Idai Cyclone and COVID-19 emergency limited the data collection. Second, there was a lack of clinical data both in this study and in the literature review, and third, we were not able to explain the high heterogeneity of the primary outcome, in relation to the systematic review with meta-analysis. Moreover, surgeons do not necessarily request examination for all appendicitis samples, and, thus, the prevalence of schistosomiasis in appendicitis may be higher than identified here. Finally, although different and more sensitive tests are available such as PCR, in our study and those identified in the review, only a histological approach was used, which is in line with the low-resource setting of these studies. Therefore, the high prevalence of schistosomiasis-related appendicitis should be interpreted with caution.

In conclusion, although schistosomiasis infection is apparently rarely associated with appendicitis, it still represents a very important global health issue. It should be considered as a possible cause of appendicitis especially in endemic areas, but, considering the global migration, also in developed countries. Finally, more effective efforts should be put in place in order to increase the prevention coverage and avoid the cascading implications for health, especially in this COVID-19 era where the majority of attention and funds are utilised to control the pandemic.

**Key Learning Points**

- Schistosomiasis is a parasitic waterborne disease affecting more than 240 million people worldwide every year, especially in sub-Saharan Africa.
- Acute appendicitis is seldom reported as schistosomal consequence, but the prevalence is underestimated.
- In our study, we reported a 13% prevalence of parasitic infection; this is 10 times higher than what was identified in the systematic review, showing a total prevalence of 1.31%.
- It will be crucial to strengthen the prevention coverage in order to avoid the cascading implications for health, especially in this Coronavirus Disease 2019 (COVID-19) era where the majority of attention and funds are used to fight the pandemic.

**Top Five Papers**

1. Colley DG, Bustinduy AL, Secor WE, King CH. Human schistosomiasis. Lancet. 2014; 383:2253–64.
2. King CH, Dickman K, Tisch DJ. Reassessment of the cost of chronic helmintic infection: a meta-analysis of disability-related outcomes in endemic schistosomiasis. Lancet. 2005;365:1561.
3. Adebamowo CA, Akang EE, Ladipo JK, Ajao OG. Schistosomiasis of the appendix. Br J Surg. 1991 Oct;78(10):1219–21.
4. Amer AS, Saad AE, Antonios SN, Hasby EA. Prevalence of Parasitic Infections in Surgically Removed Appendices: Parasitological and Histopathological Studies. Helminthologia. 2018 Jan 27;55(1):33–44.
5. Botes SN, Ibirogba SB, McCallum AD, Kahn D. Schistosoma prevalence in appendicitis. World J Surg. 2015 May;39(5):1080–3.

## Supporting information

S1 Table The quality of the studies according to the Newcastle–Ottawa assessment scale adapted for cross-sectional studies. (DOCX)

S1 Protocol Systematic review protocol for schistosomal appendicitis: Case series and systematic literature review. (DOCX)
