# Supplementary material for: Schistosomal appendicitis: Case series and systematic literature review
Source: PLoS Negl Trop Dis. 2021 Jun 24;15(6):e0009478. doi: 10.1371/journal.pntd.0009478 (PMC8224979; doi:10.1371/journal.pntd.0009478)
Supplement: S1 Table — (DOCX) [file pntd.0009478.s001.docx]

**S1 Table.** The quality of the studies according to the Newcastle - Ottawa assessment Scale adapted for cross-sectional studies.

|  | 1 | 2 | 3 | 4 | 5 | 6 | 7 | Tot |
| --- | --- | --- | --- | --- | --- | --- | --- | --- |
| Abo-Alhassan, 2016 [1] | 1 | 0 | 0 | 1 | 1 | 2 | 1 | 6 |
| Abu-Eshy, 1994 [2] | 1 | 0 | 0 | 2 | 2 | 2 | 1 | 8 |
| Adebamowo, 1991 [3] | 1 | 0 | 0 | 2 | 1 | 2 | 1 | 7 |
| Adisa, 2009 [4] | 1 | 0 | 0 | 1 | 1 | 2 | 1 | 6 |
| Ahmed, 2014 [5] | 1 | 0 | 0 | 1 | 1 | 1 | 1 | 5 |
| Ahmed, 2017 [6] | 1 | 0 | 0 | 1 | 1 | 2 | 1 | 6 |
| Al-Kraida, 1988 [7] | 1 | 0 | 0 | 1 | 0 | 2 | 1 | 5 |
| Amer, 2017 [8] | 1 | 0 | 1 | 2 | 2 | 2 | 1 | 9 |
| Badmos, 2006 [9] | 1 | 0 | 0 | 2 | 1 | 2 | 1 | 7 |
| Botes, 2015 [10] | 1 | 0 | 0 | 1 | 1 | 2 | 1 | 6 |
| Dincel, 2017 [11] | 1 | 0 | 0 | 1 | 1 | 2 | 1 | 6 |
| Duvie, 1987 [12] | 1 | 0 | 0 | 1 | 0 | 2 | 1 | 5 |
| Elfaedy, 2019 [13] | 1 | 0 | 0 | 2 | 1 | 2 | 1 | 7 |
| Hedya, 2012 [14] | 1 | 0 | 0 | 2 | 1 | 2 | 1 | 7 |
| Hodasi, 1988 [15] | 1 | 0 | 0 | 1 | 0 | 2 | 1 | 5 |
| Karatepe, 2009 [16] | 1 | 0 | 0 | 1 | 1 | 2 | 1 | 6 |
| Nandipati, 2008 [17] | 1 | 0 | 0 | 1 | 0 | 2 | 1 | 5 |
| Satti, 1987 [18] | 1 | 0 | 0 | 1 | 0 | 2 | 1 | 5 |
| Zaghlool, 2015 [19] | 1 | 0 | 0 | 1 | 1 | 2 | 1 | 6 |
| Zakaria, 2012 [20] | 1 | 0 | 0 | 2 | 1 | 2 | 1 | 7 |

**References**

1. Abo-Alhassan F, Faras F, Malek YM, Joneja M, Dhar PM. Schistosomal appendicitis in Kuwait A5-year study. Int J Surg Case Rep. 2016;28:303-309.
2. Abu-Eshy SA, Malik GM, Khan AR, Khan GM, Al-Shehri MY. Schistosomal appendicitis. Ann Saudi Med. 1995 Jul;15(4):347-9.
3. Adebamowo CA, Akang EE, Ladipo JK, Ajao OG. Schistosomiasis of the appendix. Br J Surg. 1991 Oct;78(10):1219-21.
4. Adisa AO, Omonisi AE, Osasan SA, Alatise OI. Clinicopathological review of schistosomal appendicitis in south western Nigeria. Trop Gastroenterol. 2009 Oct-Dec;30(4):230-2.
5. Ahmed SA, Mohammed U, Sanda RB, Makama J, Shehu MS, Ameh EA, Mayun AA. Schistosomiasis of the appendix in a tertiary hospital in northern Nigeria: a 22-year review. J Lab Physicians. 2014 Jan;6(1):18-21.
6. Ahmed NS, Mahmoud SF, El-Samman MK, Khalifa RMA. HISTOPATHOLOGICAL ANALYSIS OF SCHISTOSOMIASIS IN THE GASTROINTESTINAL TRACT WITH FIRST RECORD OF SCHISTOSOMAL APPENDICITIS FROM SOHAG, UPPER EGYPT. J Egypt Soc Parasitol. 2017 Apr;47(1):13-18.
7. Al-Kraida A, Giangreco A, Shaikh MU, Al-Shehri A. Appendicitis and schistosomiasis. Br J Surg. 1988 Jan;75(1):58-9.
8. Amer AS, Saad AE, Antonios SN, Hasby EA. Prevalence of Parasitic Infections in Surgically Removed Appendices: Parasitological and Histopathological Studies. Helminthologia. 2018 Jan 27;55(1):33-44.
9. Badmos KB, Komolafe AO, Rotimi O. Schistosomiasis presenting as acute appendicitis. East Afr Med J. 2006 Oct;83(10):528-32.
10. Botes SN, Ibirogba SB, McCallum AD, Kahn D. Schistosoma prevalence in appendicitis. World J Surg. 2015 May;39(5):1080-3.
11. Dincel O, Goksu M, Turk BA, Pehlivanoglu B, Isler S. Unexpected findings in the routine histopathological examinations of appendectomy specimens A retrospective analysis of 1,970 patients. Ann Ital Chir. 2017;88:519-525.
12. Duvie SO, Diffang C, Guirguis MN. The effects of Schistosoma haematobium infestation on the vermiform appendix: the Nigerian experience. J Trop Med Hyg. 1987 Feb;90(1):13-8.
13. Elfaedy O, Benkhadoura M, Elshaikhy A, Elgazwi K. Impact of routine histopathological examination of appendectomy specimens on patient management: a study of 4012 appendectomy specimens. Turk J Surg. 2019 Sep 23;35(3):196-201.
14. Hedya MS, Nasr MM, Ezzat H, Hamdy HM, Hassan AM, Hammam O. Histopathological findings in appendectomy specimens: a retrospective clinicopathological analysis. J Egypt Soc Parasitol. 2012 Apr;42(1):157-64.
15. Hodasi WM. Schistosoma appendicitis. Trop Doct. 1988 Jul;18(3):105-6.
16. Karatepe O, Adas G, Tukenmez M, Battal M, Altiok M, Karahan S. Parasitic infestation as cause of acute appendicitis. G Chir. 2009 Oct;30(10):426-8.
17. Nandipati K, Parithivel V, Niazi M. Schistosomiasis: a rare cause of acute appendicitis in the African American population in the United States. Am Surg. 2008 Mar;74(3):221-3.
18. Satti MB, Tamimi DM, Al Sohaibani MO, Al Quorain A. Appendicular schistosomiasis: a cause of clinical acute appendicitis? J Clin Pathol. 1987 Apr;40(4):424-8.
19. Zaghlool DA, Hassan AA, Ahmed MA, Faidah HS. INCIDENTAL PARASITIC INFECTIONS IN SURGICALLY REMOVED APPENDICES: A RETROSPECTIVE ANALYSIS. J Egypt Soc Parasitol. 2015 Dec;45(3):571-8.
20. Zakaria OM, Zakaria HM, Daoud MY, Al Wadaani H, Al Buali W, Al-Mohammed H, Al Mulhim AS, Zaki W. Parasitic infestation in pediatric and adolescent appendicitis: a local experience. Oman Med J. 2013 Mar;28(2):92-6.
