# Supplementary material for: Schistosomal appendicitis: Case series and systematic literature review
Source: PLoS Negl Trop Dis. 2021 Jun 24;15(6):e0009478. doi: 10.1371/journal.pntd.0009478 (PMC8224979; doi:10.1371/journal.pntd.0009478)
Supplement: S1 Protocol — (DOCX) [file pntd.0009478.s002.docx]

**Systematic Review Protocol for Schistosomal Appendicitis: Case Series and Systematic Literature Review**

**Title:** Schistosomal appendicitis: case series and systematic literature review

**Review Team:** Zacarias Mateus, Damiano Pizzol, Helder de Miranda, Anna Claudia Colangelo, Nicola Veronese, Lee Smith

**Review Question:** What is the global prevalence of Schistosomiasis related appendicitis?

**Searches:** “Schistosomiasis” OR “Bilharzia” OR “Schistosoma” OR “Schistosoma mansoni” OR “Schistosoma haematobium” OR “Schistosoma japonicum” OR “Schistosoma mekongi” OR “Schistosoma guin-eensis” OR “Schistosoma intercalatum” OR “Schistosome” OR “Blood flukes” OR “Trematode” OR “Trematoda” OR “Trematode infections” OR “Trematode worms”, AND “Appendicitis” OR “Appendectomy” OR “Appendicectomy”.

**Condition or Domain being Studied:** Schistosomiasis related appendicitis.

**Participants/ Population:** People with appendicitis.

**Exposure:** People with appendicitis**.**

**Comparator/Control:** Non-Schistosomiasis related appendicitis.

**Type of Study to be Included:** All retrospective or prospective studies reporting the prevalence of schistosomiasis in appendicitis will be included.

**Outcome:** Prevalence of people having a diagnosis of schistosomiasis in those having a diagnosis of appendicitis.

**Inclusion and Exclusion Criteria Following PICO**

Following the PICOS (participants, intervention, controls, outcomes, study design) criteria, we will included studies assessing:

P: People with appendicitis

I: None

C: None

O: Number/prevalence of schistosomiasis

S: Observational (case-control, cross-sectional).

All retrospective or prospective studies reporting the prevalence of schistosomiasis in appendicitis will be included. Studies will be excluded if they have no data on prevalence of schistosomiasis or if they are related to other gastro-intestinal disease. No language restriction will be applied.

**Measures of Effect:** Frequency of Schistosomiasis related appendicitis.

**Data Extraction:** For each eligible study, two independent investigators (NV, DP) will extract: name of the first author and year of publication, setting, sample size, mean age of the population, % of females, % acute appendectomy, % of patients with nausea, vomiting, pain in right iliac fossa, tender in right iliac fossa, abdominal guarding and fever.

**Risk of Bias Assessment:** Two independent authors (ZM, ACC) will assess the quality of studies using the Newcastle-Ottawa Scale (NOS). The NOS assigns a maximum of 9 points based on three quality parameters: selection, comparability, and outcome. As per the NOS grading in past reviews, we will grade studies as having a high (<5 stars), moderate (5-7 stars) or low risk of bias (≥8 stars).

**Strategy for Data Synthesis and Statistical Analyses:**

All analyses will be performed using Stata, version 15.0. For all analyses, a p-value less than 0.05 will be considered statistically significant.

The primary analysis will report the prevalence (%) of schistosomiasis in people having appendicitis, with its 95% confidence intervals (CIs). Heterogeneity across studies will be assessed by the I^2^ metric. Where significant heterogeneity is observed (I^2^ >50% and/or p<0.05), meta-regression analyses will be run, taking as moderators the factors stated in the data extraction in the sample as whole.

Publication bias will be assessed by visual inspection of funnel plots and using the Egger bias test. In case of publication bias, when ≥ 3 studies are available, we will use the Duval and Tweedie non-parametric trim-and-fill method to account for potential publication bias. Based on the assumption that the effect sizes of all the studies are normally distributed around the centre of a funnel plot, in the event of asymmetries, this procedure adjusts for the potential effect of unpublished (trimmed) studies.
